# Supplementary material for: Assessing Intra-Bundle Impregnation in Partially Impregnated Glass Fiber-Reinforced Polypropylene Composites Using a 2D Extended-Field and Multimodal Imaging Approach
Source: Polymers (Basel). 2024 Jul 30;16(15):2171. doi: 10.3390/polym16152171 (PMC11315057; doi:10.3390/polym16152171)

Appendix S1: Bibliometric analysis: Verification of literature gap assumptions

A non-extensive scientific mapping analysis was conducted using Scopus to filter articles related to specific keywords classified into four categories: “microscopy techniques”, “polymer matrix composites”, “analysis length scale”, and “target of analysis”. The keyword-based filtering operations in Scopus, along with additional filtering conditions, identified 316 scientific journal articles covering the period from 2018 to 2024. More details about the Scopus filtering instructions are provided in Table S1.b in Appendix S1 of the supplementary materials. The corresponding abstracts, author-set keywords, and indexed keywords were exported in .ris format. The open-source software VOSviewer was used to filter and analyze the set of identified articles (Refer to Table S1.c in Appendix S1 of the supplementary materials). This software was chosen for its simplicity of use, which is consistent with preliminary research exploring numerous data relationships [22]. The data analysis concept is based on mapping bibliographic data using a local moving algorithm, which identifies relationships within networks of publications via keyword co-occurrence. For instance, a link connects two keywords appearing simultaneously in the same scientific articles, and the thickness of interconnections is related to the number of occurrences of keyword pairs through the .ris file exported from the identified list of articles in Scopus. More detailed information about VOSviewer as open access software can be found in [23]. A threshold of co-occurrence levels was set to eight (meaning a minimum co-occurrence level of eight to consider the keyword significant) to facilitate reading and analysis of the generated maps illustrated in Fig.S1.a and Fig.S1.b in Appendix S1 of the supplementary materials.

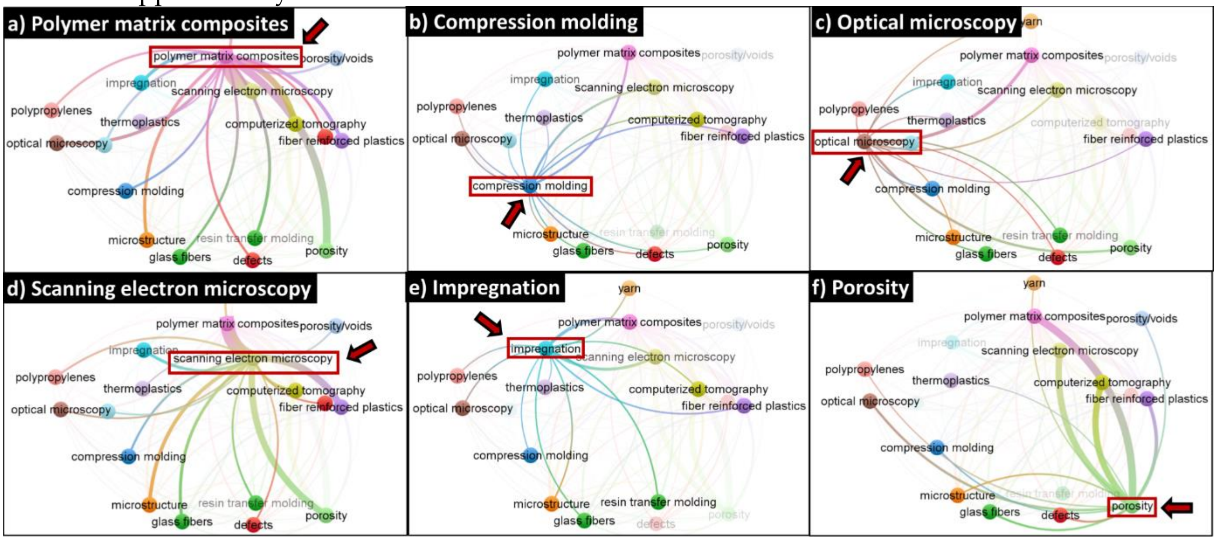

Figure S1.a VOSviewer-based scientific maps focusing on a Scopus dataset of articles. The rectangular form and the arrow indicate the central keyword in the illustrated co-occurrence networks.

As illustrated in Fig.S1.a-b, studies focusing on “polymer matrix composites” and “compression molding” frequently report the use of “scanning electron microscopy” and “optical microscopy”. Corresponding analyses were related to either “impregnation”, “porosity” or “defects”. However, there is no indication of “multimodality” between these characterization techniques with respect to the considered co-occurrence threshold level. When centering the analysis on the keywords “optical microscopy” and “scanning electron microscopy,” maps in Fig.S1.c-d show interconnections related to keywords including “microstructure”, “defects”, “porosity” and “impregnation”, with a higher reliance on SEM compared to OM. This is evident from the occurrence weights affecting inter-connections within studies related to “polymer matrix composites”, “fiber-reinforced composites”, and “porosity”. Mapped networks in Fig.S1.e-f are focused on keywords “impregnation” and “porosity” which are semantically antagonistic and seem to be connected to the same cloud of detected keywords, with “porosity” network of connection appearing more dense which reflects a higher degree of interest from the scientific community compared to “impregnation” within the limit of the identified articles. The results obtained from this broad scientific mapping can provide more

insight about the significance of the identified gaps in literature which are reflected by the absence of "multimodality" and "fluorescence microscopy" in context of polymer matrix composites.

Table.S1.b. Scopus search keywords and applied filtering criteria.

Research: Title/Abstract/Keywords
*(**TITLE-ABS-KEY** ("pmc" OR "cfrp" OR "gfrp" OR "tpc" OR "afrp" OR "frp" OR "polymer matrix composites" OR "thermoplastic matrix composites" OR "glass fiber reinforced polymer" OR "carbon fiber reinforced polymer" OR "glass fiber reinforced composite" OR "carbon fiber reinforced composite")*
***AND (TITLE-ABS-KEY** ("om" OR "sem" OR "µct" OR "optical microscopy" OR "scanning electron microscopy" OR "computed tomography" OR "fluorescence microscopy" OR "polarized light microscopy") OR ABS ("micro-scale" OR "meso-scale" OR "macro-scale"))*
***AND (TITLE-ABS-KEY** ("porosity" OR "porosity quantification" OR "porosity distribution" OR "porosity location" OR "porosity shape" OR "porosity size" OR "void" OR "residual porosity"))*
***OR (TITLE-ABS-KEY** ("impregnation" OR "degree of impregnation")*
***OR (TITLE-ABS-KEY** ("multi-modal" OR "multimodal" OR "multimodality"))*
Filtering: Years
***AND** (PUBYEAR > 2018 AND PUBYEAR < 2024)*
Filtering Keywords
AND (LIMIT-TO (EXACTKEYWORD , "Polymer Matrix Composites"))
OR (LIMIT-TO (EXACTKEYWORD , "Scanning Electron Microscopy"))
OR (LIMIT-TO (EXACTKEYWORD , "Porosity") OR LIMIT-TO (EXACTKEYWORD , "Computerized Tomography"))
OR (LIMIT-TO (EXACTKEYWORD , "Fiber Reinforced Plastics"))
OR (LIMIT-TO (EXACTKEYWORD , "Carbon Fiber Reinforced Plastics"))
OR (LIMIT-TO (EXACTKEYWORD , "X-ray Computed Tomography"))
OR (LIMIT-TO (EXACTKEYWORD , "Reinforced Plastics"))
OR (LIMIT-TO (EXACTKEYWORD , "Laminated Composites"))
OR (LIMIT-TO (EXACTKEYWORD , "Morphology"))
OR (LIMIT-TO (EXACTKEYWORD , "Composite Materials"))
OR (LIMIT-TO (EXACTKEYWORD , "Microstructure"))
OR (LIMIT-TO (EXACTKEYWORD , "Nondestructive Examination"))
OR LIMIT-TO ((EXACTKEYWORD , "Impregnation"))
OR (LIMIT-TO (EXACTKEYWORD , "Composites"))
OR (LIMIT-TO (EXACTKEYWORD , "Defects"))
OR LIMIT-TO (EXACTKEYWORD , "CFRP") OR LIMIT-TO (EXACTKEYWORD , "Optical Microscopy")
OR LIMIT-TO (EXACTKEYWORD , "Void Fraction")
OR LIMIT-TO (EXACTKEYWORD , "Ultrasonic Testing") OR LIMIT-TO (EXACTKEYWORD , "Polymer-matrix Composites (PMCs)")
OR LIMIT-TO (EXACTKEYWORD , "Carbon Fiber Reinforced Polymer")
OR LIMIT-TO (EXACTKEYWORD , "Fiber Reinforced Materials")
OR LIMIT-TO (EXACTKEYWORD , "SEM")
OR LIMIT-TO (EXACTKEYWORD , "Polymer-matrix Composites")
OR LIMIT-TO (EXACTKEYWORD , "Manufacturing Defects")
OR LIMIT-TO (EXACTKEYWORD , "Pore Size")
OR LIMIT-TO (EXACTKEYWORD , "Voids")
OR LIMIT-TO (EXACTKEYWORD , "X Ray Micro-computed Tomography")
OR LIMIT-TO (EXACTKEYWORD , "High Resolution Transmission Electron Microscopy")
OR LIMIT-TO (EXACTKEYWORD , "Microscopy, Electron, Scanning")
OR LIMIT-TO (EXACTKEYWORD , "Polymer-matrix Composite")
OR LIMIT-TO (EXACTKEYWORD , "Computed Tomography")
OR LIMIT-TO (EXACTKEYWORD , "Porosity/voids"))
Filtering Countries
AND (LIMIT-TO (AFFILCOUNTRY , "United States")
OR LIMIT-TO (AFFILCOUNTRY , "United Kingdom")
OR LIMIT-TO (AFFILCOUNTRY , "Germany")

OR LIMIT-TO (AFFILCOUNTRY , "France")
OR LIMIT-TO (AFFILCOUNTRY , "Japan")
OR LIMIT-TO (AFFILCOUNTRY , "South Korea")
OR LIMIT-TO (AFFILCOUNTRY , "Italy")
OR LIMIT-TO (AFFILCOUNTRY , "Austria")
OR LIMIT-TO (AFFILCOUNTRY , "Turkey")
OR LIMIT-TO (AFFILCOUNTRY , "Canada")
OR LIMIT-TO (AFFILCOUNTRY , "Saudi Arabia")
OR LIMIT-TO (AFFILCOUNTRY , "Australia")
OR LIMIT-TO (AFFILCOUNTRY , "Spain")
OR LIMIT-TO (AFFILCOUNTRY , "Netherlands")
OR LIMIT-TO (AFFILCOUNTRY , "Belgium")
OR LIMIT-TO (AFFILCOUNTRY , "Finland")
OR LIMIT-TO (AFFILCOUNTRY , "Portugal")
OR LIMIT-TO (AFFILCOUNTRY , "Sweden"))
Filtering document type
AND (LIMIT-TO (DOCTYPE , "ar"))
Filtering language
AND (LIMIT-TO (LANGUAGE , "English"))

Table.S1.c List of retained keywords after applying an occurrence threshold of 8 in VOSviewer.

id	*keyword*	*occurrences*	*total link strength*
590	compression molding	11	11.00
613	computerized tomography	83	76.00
770	defects	19	19.00
1114	fiber reinforced plastics	68	64.00
1323	glass fibers	27	27.00
1519	impregnation	19	19.00
1913	microstructure	24	24.00
2116	optical microscopy	16	16.00
2344	polymer matrix composites	166	154.00
2386	polypropylenes	11	11.00
2413	porosity	82	81.00
2422	porosity/voids	10	10.00
2603	resin transfer molding	15	15.00
2657	scanning electron microscopy	146	138.00
3051	thermoplastics	8	8.00
3225	void contents	8	8.00
3230	void fraction	16	14.00
3328	yarn	12	12.00

Figure.S1.d VOSviewer-generated map and network of connections between co-occurring keywords.

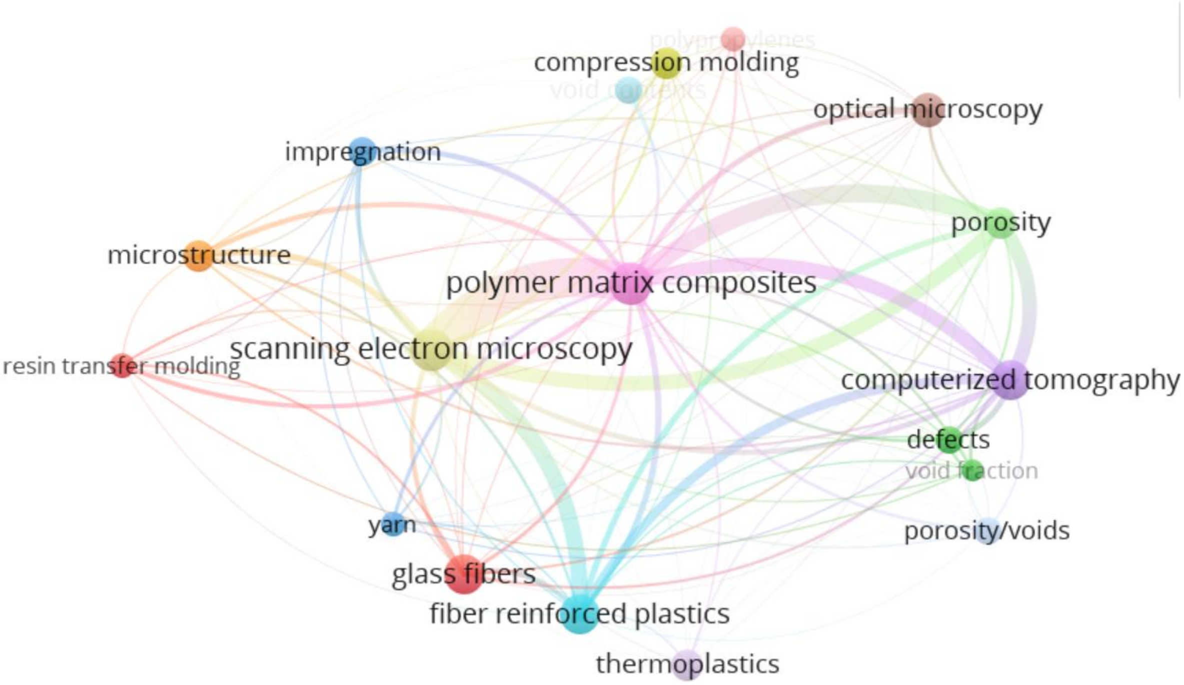

Appendix S2: Full-Scale, Extended-Field Acquisitions and Image Analyses

Extended-field acquisition is a valuable technique that involves creating a grid of elementary images, which are subsequently stitched together to provide an expansive and detailed view of the microstructure. This approach relies on collecting detailed and high-resolution local images that partially overlap. In this study, the technique was specifically employed to include at least six full bundle cross-sections and the entire thickness of the composite plate, ensuring comprehensive coverage. The resolution was carefully calibrated according to the magnification levels of the employed imaging methods. A standard magnification of X80 was utilized with the Zeiss microscope to clearly visualize individual fibers, while a higher magnification of X200 was applied in SEM analyses using the BED-C mode to capture finer structural details. This strategy ensures that both macroscopic and microscopic features of the composites are captured with clarity and precision, facilitating a thorough investigation of the material's properties.

Table S2.1. Overview of key parameters about extended-field acquisitions and resulting output images.

**Plate**	**Acquisition Technique**	**Grid (Col, Row)**	**Tile Size (pixel)**	**Pixel Size (pixel/µm)**	**Raw Image (pixel)**	**Raw Image (MB)**	**Final Output Dimensions (pixel)**	**Final Output Image (MB)**
Cr_0%	SEM : Magnif.: X200 Mode : BED-C Voltage : 15kV	850 (34; 25)	1280×960 (8-bit)	2.13	32760×15062 (8-bit)	471	17088×7776 (8-bit)	127
	PLM : Magnif.: X80 White light : 82% Exposure: 100 ms	247 (19; 13)	1812×1216 (RGB)	1.936	17943×8516 (RGB)	583	17088×7776 (RGB)	507
	FM Magnif.: X80 UV light: 25% Exposure: 80 ms	247 (19; 13)	1812×1216 (RGB)	1.936	177542×8413 (RGB)	570	17088×7776 (RGB)	507
Cr_30%	SEM : Magnif.: X200 Mode : BED-C Voltage : 15kV	348 (29; 12)	1280×960 (8-bit)	2.13	32979×9674 (8-bit)	304	16992×5952 (8-bit)	96
	PLM : Magnif.: X80 White light : 82% Exposure: 100 ms	190 (19; 10)	1812×1216 (RGB)	1.936	17776×6657 (RGB)	451	16992×5952 (RGB)	386
	FM Magnif.: X80 UV light: 25% Exposure: 80 ms	191 (19; 10)	1812×1216 (RGB)	1.936	17680×6611 (RGB)	446	16992×5952 (RGB)	386
Cr_41%	SEM : Magnif.: X200 Mode : BED-C Voltage : 15kV	319 (29; 11)	1280×960 (8-bit)	2.13	30726×9563 (8-bit)	280	16928×4880 (8-bit)	79
	PLM : Magnif.: X80 White light : 82%	171 (19; 9)	1812×1216 (RGB)	1.936	17761×6044 (RGB)	409	16928×4880 (RGB)	315

	Exposure: 100 ms							
	FM<br>Magnif.: X80<br>UV light: 25%<br>Exposure: 80 ms	172 (19; 9)	1812×1216 (RGB)	1.936	17806×6050 (RGB)	411	16928×4880 (RGB)	315

Appendix S3: Individual bundles extracted from Cr_0%, Cr_30% and Cr_41% samples based on SEM raw data, normalized individual distances between single GF, bundle contours, raw FM images and classified pixels of FM images.

Fig.S3.a Dataset of 0° oriented bundles extracted from plate Cr_0%

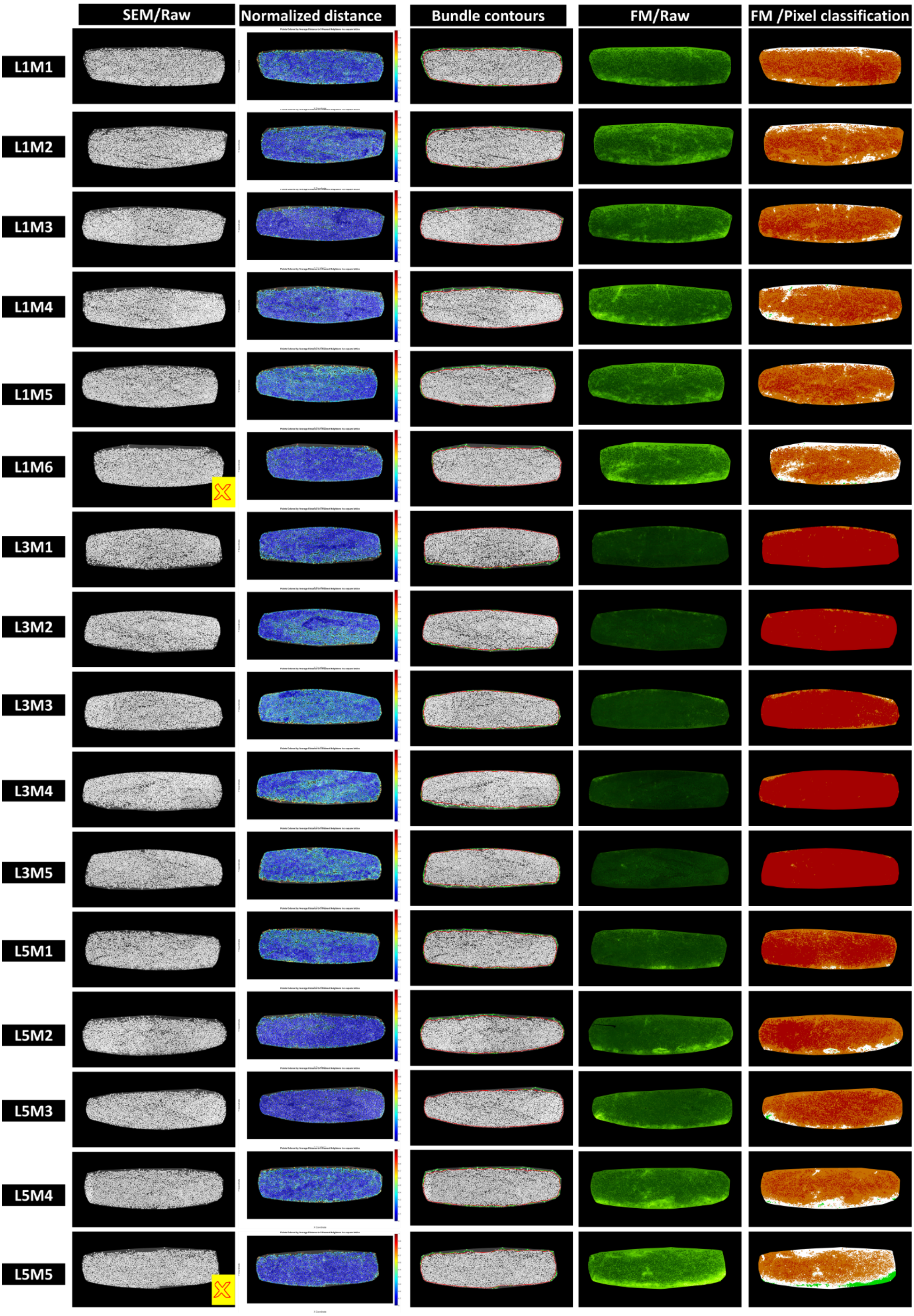

Fig.S3.b Dataset of 0° oriented bundles extracted from plate Cr_30%

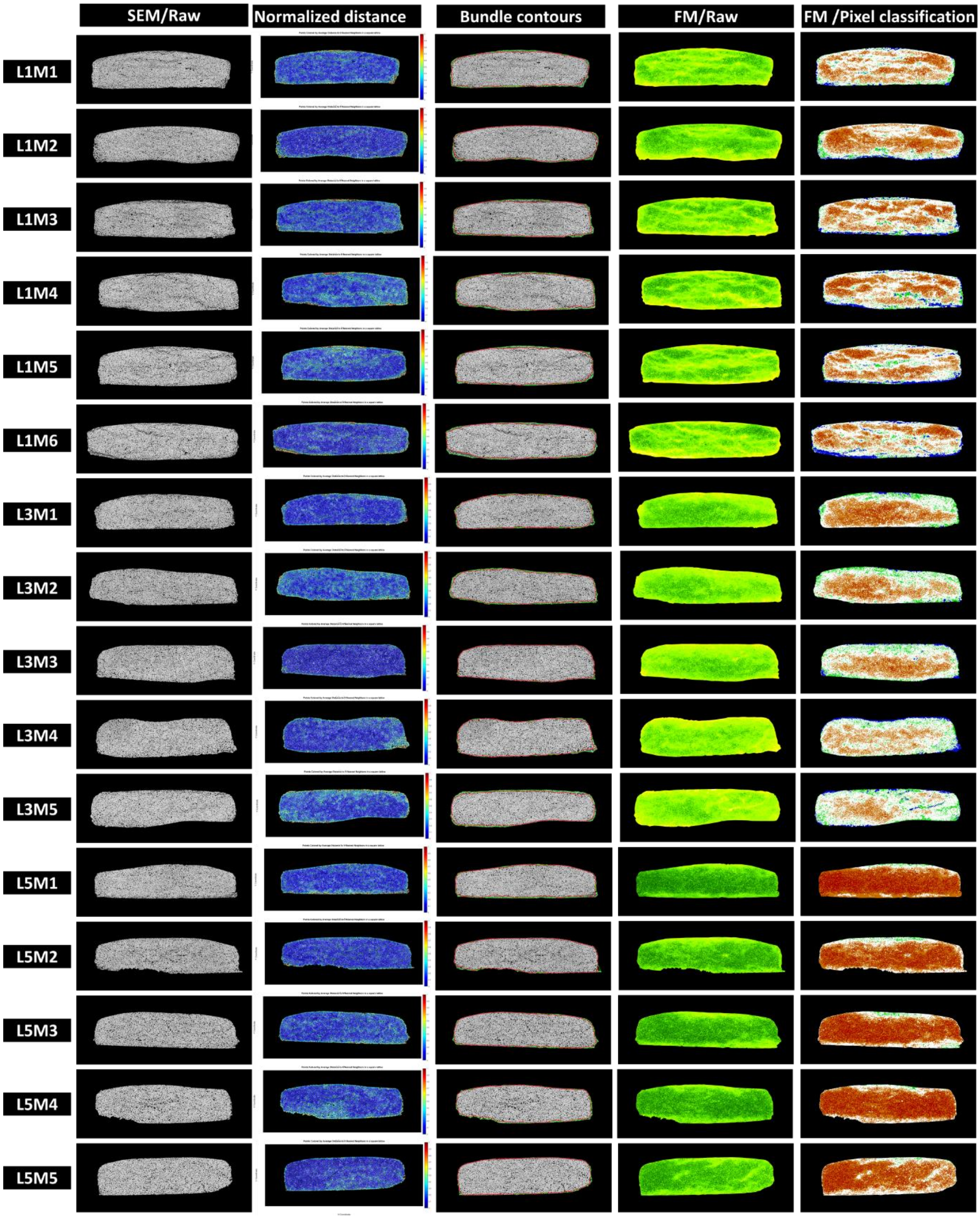

Fig.S3.c Dataset of 0° oriented bundles extracted from plate Cr_41%

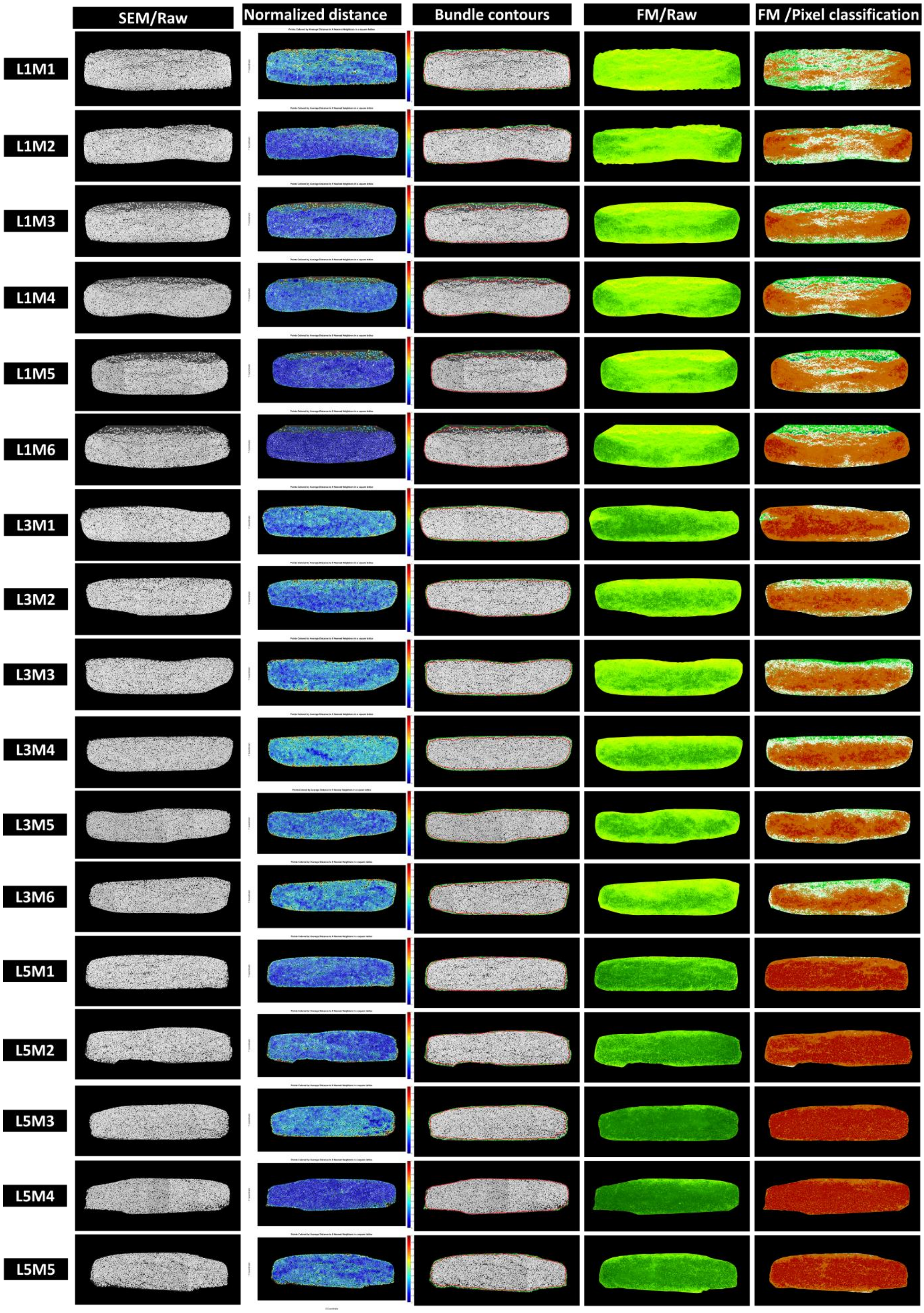

Appendix S4: Histogram analysis of gray levels of macro-scale SEM images of Cr_0%, Cr_30% and Cr_41%.

Histograms of grey levels of the stitched macro-scale SEM images from Fig.7.g-I are provided in Fig.S4 The corresponding data are provided in Fig.S4. The three histograms of grey levels, corresponding to images in Fig.7.g-I, show grey level peaks above 200, corresponding primarily to pixels appearing in white which are principally coincident with pixels corresponding to GF and those at the edge of single GF having light grey levels. In the cases of Cr_30% and Cr_41% samples, these peaks indicate the superposition of at least two Gaussian distributions of pixels. This observation can be associated either to pixels at the interface between GF and their surrounding polymer rich environment (either PP or mounting epoxy), leading to high grey levels with lower intensity than pixels located at the centre of single GF, or due to the signal drift associated with extensive SEM acquisitions of single tiles (see Table S2, in Appendix S2, in supplementary materials), causing local shifts in the mean of Gaussian peaks towards lower value grey levels. This drift effect is more pronounced for Gaussian peaks of polymeric components, particularly PP and epoxy resin mount, which show peaks between approximately 25 and 150 grey levels. All three histograms reveal more than two distinct Gaussians, confirming grey level changes due to signal drift. As the authors used stitched raw SEM tiles (without any image correction or filtering) the use of a global grey level thresholds to separate PP and epoxy pixels is not possible. These observations have led the authors to employ random forest classifiers based on Illastik for more localized and objective SEM image segmentation. Despite these efforts, the higher precision in pixel segmentation is attributed mainly to GF rather than the differentiation of PP and resin mount pixels, as confirmed by the output images of segmented SEM macro-scale images provided in Fig.8.d-f.

Fig.S4: Histograms of grey levels of the stitched macro-scale SEM images from Fig.7.g-I

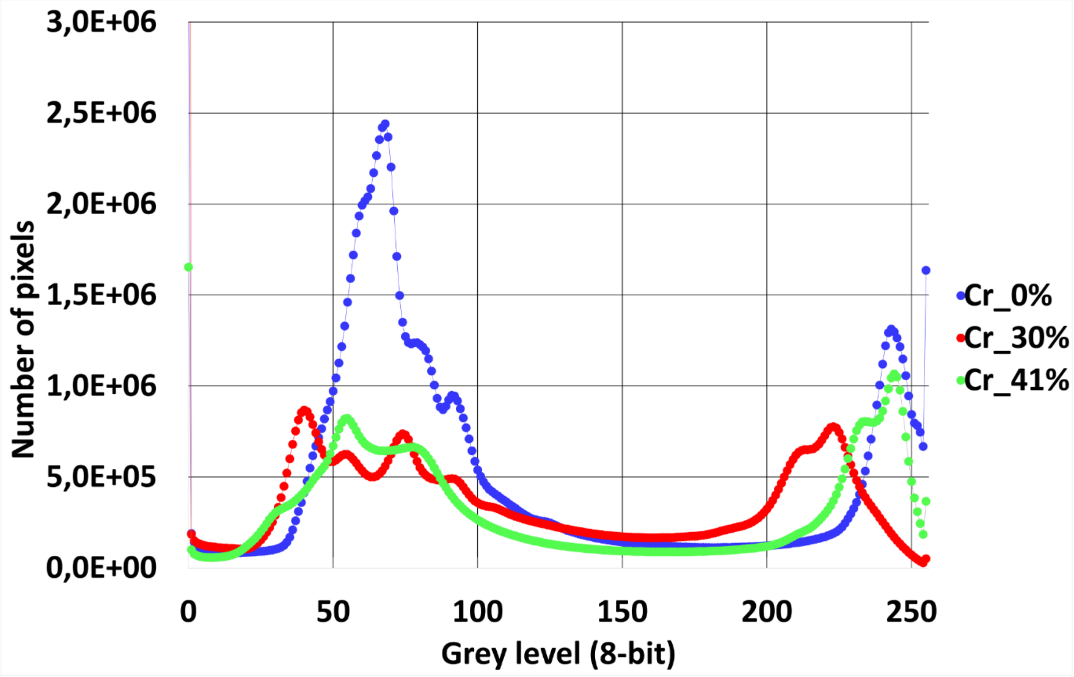

Supplement: Supplementary file 1 [file polymers-16-02171-s001.zip › polymers-3099949-supplementary.pdf]
